# Islands contribute disproportionately high amounts of evolutionary diversity in passerine birds

**DOI:** 10.1038/ncomms9538

**Published:** 2015-10-05

**Authors:** Knud A. Jønsson, Ben G. Holt

**Affiliations:** 1 Department of Life Sciences, Imperial College London, Silwood Park Campus, Ascot SL5 7PY, UK; 2 Department of Life Sciences, Natural History Museum, Cromwell Road, London SW7 5BD, UK

## Abstract

Island systems generally have fewer species than continental areas due to their small size and geographical isolation. Low island diversity reduces the possibility of exportation of island lineages and island systems are not thought to have a major influence on the build-up of continental diversity. However, the view that islands represent the end of the colonization road has recently been challenged and islands do represent the origin of some specific continental lineages. Here we assess the net contribution of island systems to global diversity patterns of passerine birds, using a complete phylogeny (5,949 species), biogeographical regionalization and null-model comparisons. We show that, in contrast to major continental regions, island regions export relatively more evolutionary lineages than would be expected based on current distributional patterns. This result challenges a central paradigm in island biogeography and changes our perception of the relative importance of islands for the build-up of global diversity.

## Abstract (web summary)

Islands generally have fewer species than continental areas and are not thought to contribute significantly to continental diversity. Here, the authors show islands can be more dynamic and export many more evolutionary lineages than expected based on contemporary diversity patterns.

## Graphical abstract

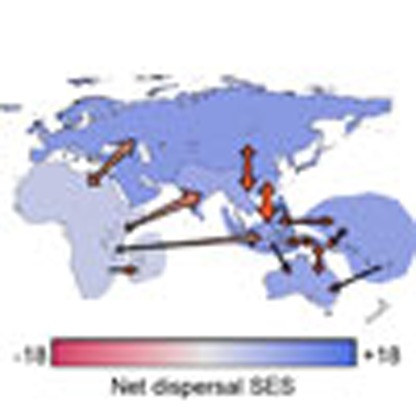

Island systems have played a disproportionally large role in the study of the evolution, redistribution and maintenance of biological diversity. Contributions by Darwin^1^ and Wallace^2^,^3^ formed the antecedent work for the formulation of the equilibrium theory of island biogeography by MacArthur and Wilson^4^,^5^ in which they predicted species richness and turnover on islands as an emergent equilibrium between immigration and extinction. Today the focus on island systems as model systems for ecological and evolutionary research is stronger than ever^6^ despite the common assumption that insular faunas are depauperate and stem from a simple one-way, downstream flow of colonists from continents^4^,^5^,^7^,^8^. However, this assumption has been challenged recently in a series of studies demonstrating that islands may in some cases represent sources for continental diversity^9^,^10^,^11^.

Here we use complete global distributional^12^ and phylogenetic data^13^ for passerine birds (5,949 species), biogeographical regionalizations, ancestral state reconstructions and a null-model-based analytical approach to examine the spatial dynamics of species distributions with a particular emphasis on the exchange of evolutionary lineages between biogeographical regions and the relative role of continental versus insular regions in contributing to global diversity. We show that islands not only contribute to continental diversity but also contribute relatively more to global diversity than expected based on the current distributions of passerine species.

## Results

### Comparing locations and bioregionalization

**Figure 1:**
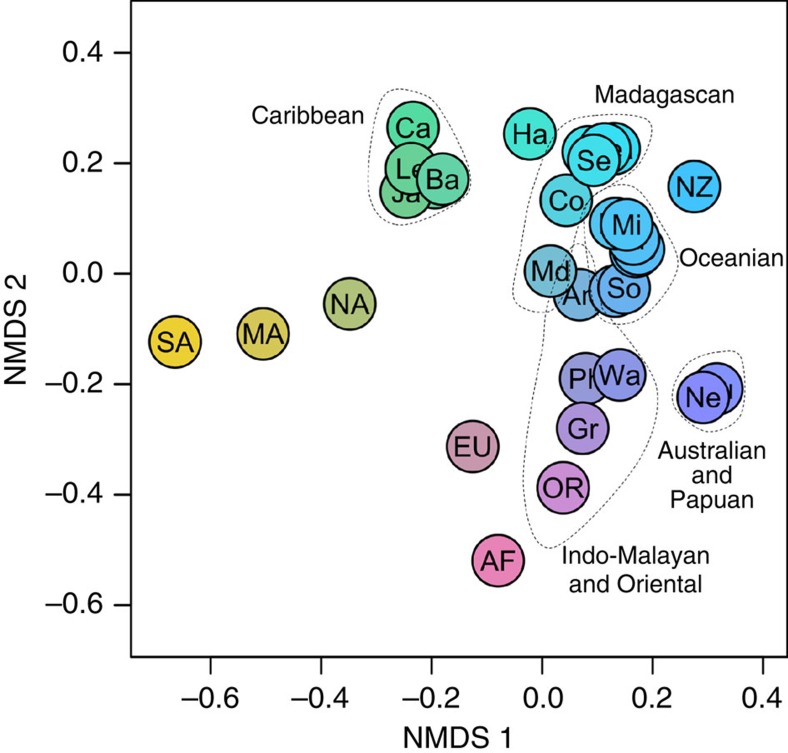
Ordination of global passerine bird phylo-betadiversity. Non-metric multidimensional scaling (NMDS) ordination of pairwise Sørensen phylo-betadiversity scores between 32 major global passerine communities. Dotted lines indicate regions that have been clustered together in subsequent clustering analysis. AF, Africa; An, Andaman and Nicobar Islands; Au, Australia; Ba, Bahamas; Bi, Bismarck Islands; Ca, Cayman Islands, San Andres and Providencia; Co, Comores Islands; Cu, Cuba; EU, Eurasia; Fi, Fiji; Gr, Greater Sunda Islands; Ha, Hawaii; Ja, Jamaica, Hispaniola and Puerto Rico; Le, Lesser Antilles; MA, Middle America; Ma, Mauritius; Md, Madagascar; Mi, Micronesia; NA, North America; NC, New Caledonia; Ne, New Guinea; NZ, New Zealand; Va, Vanuatu; OR, Oriental; Ph, Philippines and Palawan; Po, Polynesia; Re, Reunion; SA, South America; S., South Seychelles; Se, Seychelles; So, Solomon Islands; Wa, Wallacea.

**Figure 2:**
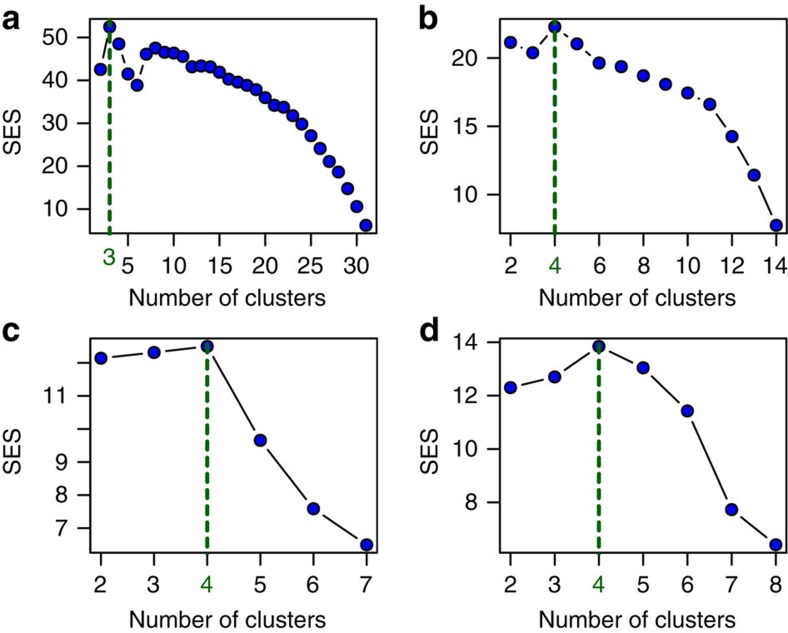
Standardized effect sizes for phylo-betadiversity clustering of global passerine assemblages. Standardized effect sizes for phylo-betadiversity clustering results of global bird community data, (**a**) all locations, (**b**) within Indo-Pacific realm, (**c**) within New World realm and (**d**) within Old World realm.

The initial stage of our analysis was to establish biogeographical regions within current passerine distributions, to use as analytical units for our main analysis. On the basis of a list of all 5,949 passerine bird species distributed across 32 locations according to the International Ornithological Committee^12^ and a recent complete bird phylogeny^13^, we established phylogenetic similarities and differences among global passerine assemblages (Fig. 1) and used this information in conjunction with null randomization to detect the most suitable biogeographical groupings for these regions (Figs 1 and 2). The 32 original locations were divided into three realms, with each of these realms being divided into four regions (the number of regions per realm being consistent purely by coincidence (Fig. 2)). Following this result we further split the cluster containing Australia and New Guinea, and also the cluster containing the Oriental and the Indo-Malayan Islands, to allow for comparisons of insular and continental systems. This resulted in 14 global regions for passerine birds, which were used for ancestral character state reconstruction.

### Detecting local speciation, import and export

On the basis of an ancestral character state analysis, 10,415 of 11,785 available nodes were both assigned to a regional character state and had an ancestor that was assigned to a regional character state (based on a 75% probability threshold). The assigned regional character state internode branches were then used to identify specific biogeographical/evolutionary events: either ‘*in situ* speciation' (cases in which an ancestral node is assigned to the same region as its descendent node) or ‘evolutionary transitions' (cases in which an ancestral node is assigned to a different region than its descendent node). Evolutionary transitions involving a focal region receiving a descendent node from an ancestral node in a different region were referred to as ‘import' events and reverse situations were referred to as ‘export' events. The balance between the number of import and export events for a region was referred to as ‘net dispersal'. The ancestral reconstruction facilitated the assignment of 10,579 biogeographical evolutionary events; this number is slightly higher than 10,415 due to a few nodes being assigned to regional character states representing combined regions. The majority of events represented *in situ* speciation events, 9,384 compared with 1,195 transition events. The total numbers of transition events and *in situ* speciation events per region is given in Table 1.

**Figure 3:**
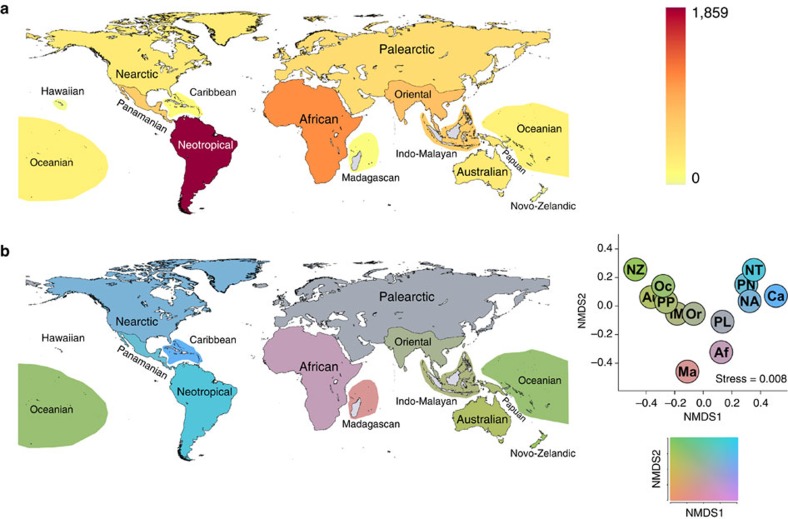
Global diversity patterns for passerine birds. Current global diversity patterns for passerine birds based on distributions obtained from the International Ornithological Committee World Bird List; (**a**) alpha diversity with highest species diversity coloured red and (**b**) betadiversity with the Sørensen betadiversity ordination to the right of the map and the Hue-Chroma-Luminance colour scheme below. Note that the Hawaiian Islands could not be included in the colour scheme for **b** because they could not be included in the ordination of pairwise Sørensen index values.

**Figure 4:**
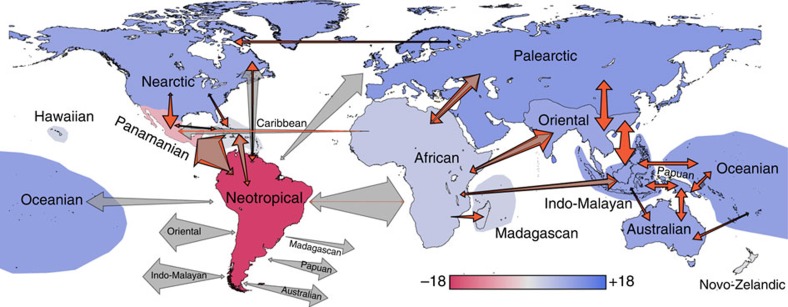
Global evolutionary dispersal map for passerine birds. Map showing each region coloured according to net dispersal (that is, export of species minus import of species) with blue indicating more than expected export and red indicating more than expected import (based on standardized effect sizes, which compare empirical values with null expectations based on current distribution patterns). Shaded arrows indicate expected dispersal according to the null models and orange arrows indicate the empirical dispersal. The size of the arrows is proportional to the numbers of evolutionary lineages moving in each specific direction. Only the most significant (that is, highest standardized effect sizes) pairwise comparisons are shown.

**Figure 5:**
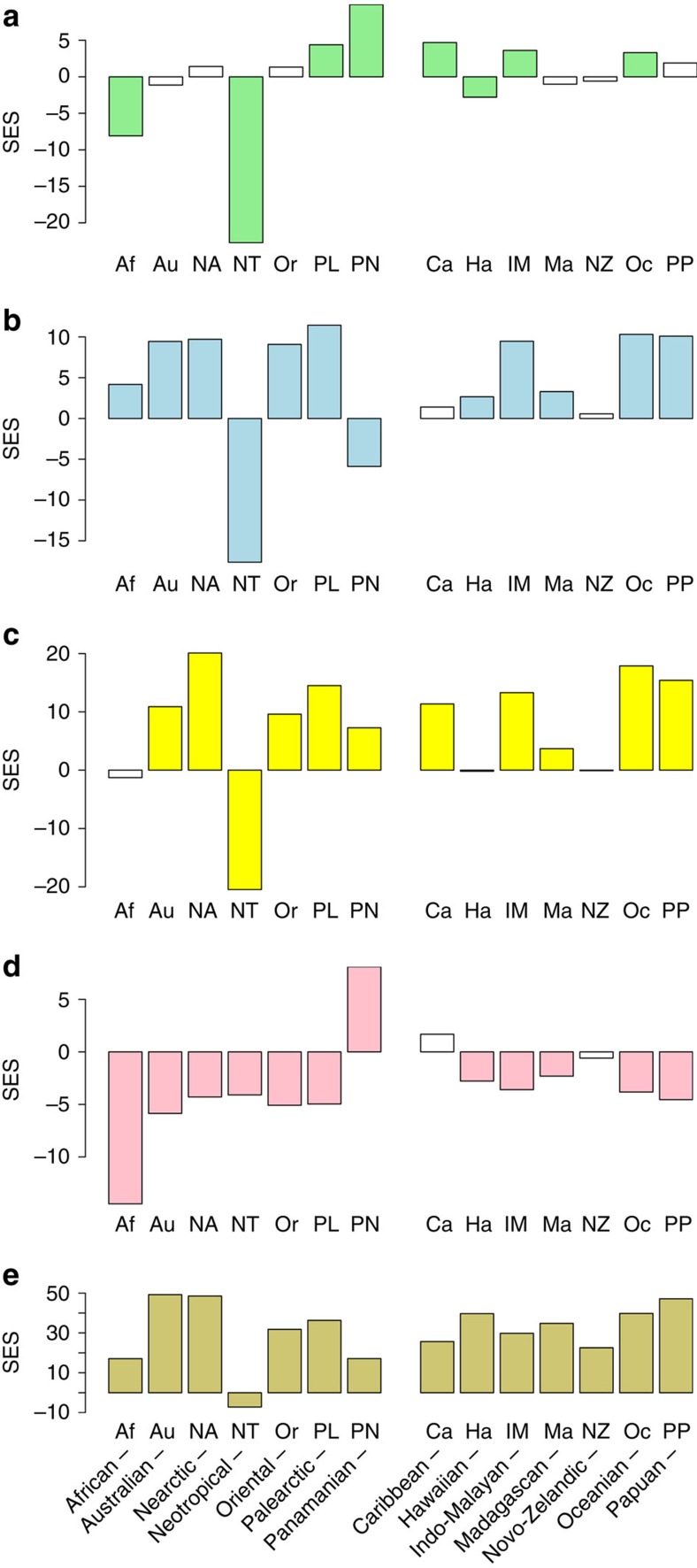
Regional dispersal and speciation relative to the expected. Bar plots indicate various dispersal metrics compared with the null expectations derived from randomizations constrained to account for existing diversity patterns (that is, standardized effect sizes). From the top: (**a**) total dispersal (import and export combined), which is an indication of how dynamic the region is, (**b**) net dispersal (export of species minus import of species), which is an indication of whether the region is overall a source or a sink, (**c**) export, (**d**) import and (**e**) diversification within regions.

As expected, due to current distributional patterns (Fig. 3), large continental regions (for example, the Neotropical and the African regions) account for the majority of transitions (Fig. 4, red arrows). However, the extent of these continental transitions is often considerably lower than null expectations based on current distributional patterns (Fig. 4, grey arrows). Our region-specific standardized effect sizes (SES's), which standardize observed values by null expectations (via SES values for which a value higher than 1.96 or lower than −1.96 is significant, assuming a normal distribution of null results), show a rather different picture to the raw observations, with island regions contributing much higher amounts of evolutionary diversity than would be expected based on their low diversity and isolation (Fig. 5 and Supplementary Fig. 1).

## Discussion

Our analyses demonstrate that extensive archipelagos (for example, the Indo-Malayan, the Oceanian and the Caribbean regions) are evolutionarily highly connected to other regions, with no evidence of reduced export of the evolutionary lineages they generate (Figs 4 and 5a). On the contrary, the Indo-Malayan and the Oceanian archipelagos are clear net exporters of diversity and thus act as sources of evolutionary lineages (Fig. 5b). Even the geographically isolated Novo-Zelandic archipelago shows no evidence of being an evolutionary sink and produces roughly the expected number of dispersal events, whereas the Hawaiian islands do show reduced dispersal but mainly with regards to import (Fig. 5a–d). Continental regions, on the other hand, show an inconsistent pattern. Some continents are much more poorly connected to the rest of the world than their current species richness would suggest (for example, Neotropics; Fig. 5a, see Simpson^14^ for an account of this ‘splendid isolation'), whereas others are very well connected (for example, Palaearctic, Oriental and African; Fig. 5a). In addition, some continents show much higher levels of export than import, whereas others show the opposite pattern (Fig. 5b).

The most dynamic regions according to our analysis are the Indo-Pacific island regions (Indo-Malayan and Oceanian), the Caribbean, the Panamanian and the Palaearctic, which all show higher than expected levels of total dispersal (Fig. 5a). In stark contrast to the raw empirical values, the large tropical continental regions are the least dynamic relative to null expectations (Figs 4 and 5a). Patterns are inconsistent across continental regions and the Nearctic, the Palaearctic, the Oriental and the Australian show higher than expected net dispersal and also high *in situ* speciation. The Neotropical region may have high levels of species richness, potentially reflecting its size, as well as its geological and environmental history but it is a particularly isolated area for passerine birds: importing less than expected and exporting much less than expected. Similarly, the standardized effect sizes for African net dispersal is also considerably lower than for some archipelagos. The high total dispersal of the Panamanian region is attributable to a high exchange level (both high export and import), with the Caribbean, the Nearctic and the Neotropics, a result that may be directly influenced by the geological history of all of these regions. However, the net balance of dispersal is predominantly to, rather than from, the Panamanian region, which therefore acts as a net sink for evolutionary lineages (Fig. 5b). Conversely, the total dispersal of many other regions, including all the island regions across the Indo-Pacific and in the Caribbean, is driven mainly by high export and these areas are therefore true source areas of evolutionary diversity. The complex geological history of the Indo-Malayan region complicates interpretation of the dispersal results for this region because some parts were physically connected to the Oriental during the Pliocene, and there is therefore potential for some events to represent vicariance rather than dispersal. However, this region also shows significantly high dispersal to the African, the Australian, the Oceanian and the Papuan regions, and is clearly an important exporter of evolutionary diversity. The results for the Caribbean and the Oceanian regions are unlikely to have been influenced by vicariance since the Pliocene.

From our analyses it is evident that all regions generate evolutionary diversity and this diversity is often re-distributed to other regions. Taken together these results suggest that both islands and continental regions can act as macroevolutionary sources, or as ‘centres of origin'^15^, whereas some continental regions appear to be ‘centres of accumulation'^15^,^16^,^17^,^18^, generating less of their own evolutionary diversity than expected due to the number of extant species within them. However, due to variation in current species richness across the regions, continental regions inevitably generate more diversity than islands in terms of absolute numbers. The Neotropics show some features reminiscent of a sink (that is, low export, but also low import), which is counter-intuitive because this area is home to the highest number of passerine bird species, but our analysis has factored in contemporary diversity and considers the contribution of regions towards the evolution of present-day diversity. On this basis, regions within the New World appear to be poorly interconnected relative to those found in the Old World. It has been suggested that the east–west axis of Eurasia led to advantages for the first humans because biological and cultural exchange took place largely within similar climatic niches^19^. It is plausible that, in a similar fashion, this east–west axis has facilitated relatively high bird dispersal over evolutionary timescales, whereas dispersal in the New World was only really extensive between the tropical South America and the tropical Middle America via the narrow Panama land bridge, which connected Central and South America since the mid-Miocene^20^.

While phylogenies provide insights into temporal diversification patterns of lineages, a high level of uncertainty remains about the diversification process owing to potentially unknown variable rates of extinction across the tree^21^,^22^ and among geographical regions. Consequently, determining the sequential arrival of particular clades in particular regions depends to some degree on the assumption that extinction has been on average the same in all regions and non-random extinctions of individual lineages and clades^23^ could bias results. However, we see no plausible reason our main finding regarding the evolutionary dynamic nature of island systems might be an artefact of such patterns. Island systems are in fact considered to have high extinction rates, in which case it would be more likely that continental taxa might outlive species with island-based ancestors than island taxa having outlived species with continental ancestors. We also note that while our pattern could be driven by uncertainty of ancestral state reconstruction towards the root of the tree, the majority of both evolutionary transition events (89.4%) and *in situ* speciation events (77.8%) have taken place since the beginning of the Pliocene (5.33 million years ago).

The last half century of research has continuously elaborated on the legacy of MacArthur and Wilson^4^,^5^ and the notion that island diversity is established exclusively through dispersal from biologically diverse continental biotas. Our study, however, provides strong evidence that islands not only contribute to continental diversity but also can contribute proportionately more to global diversity than expected based on the number of species that inhabit them. It has been suggested that the dynamics of extensive island systems may allow for sustained high net diversification rates in insular settings, and it appears plausible that this increased diversification rate can result in new evolutionary lineages that are exported to other regions^24^. This changes our view on island biogeography markedly, in the sense that islands when considered as insular ecological and evolutionary clusters rather than as single entities act as important macroevolutionary sources. While future analyses will establish whether other globally distributed taxonomic groups show similar patterns, our results endorse the high level of research focusing on island systems and suggest they are highly relevant to the evolution of life across all terrestrial regions.

## Methods

### Comparing locations

Initially, we considered all possible pairwise comparisons of the locations in our distribution data set; focusing on the phylogenetic composition of the passerine communities in these locations. We quantified these comparisons using the phylogenetic version of the Sørensen index: ‘PhyloSor'^25^. This metric was preferred to phylo-betadiversity metrics previously used previous bioregionalization analysis, such as phylobeta_sim_ (ref. 26), as is the PhyloSor metric is influenced by diversity gradients, which are highly relevant to this study.

We also mapped global species richness and betadiversity (Sørensen) for passerine birds to provide baseline information for our main analysis. Sørensen betadiversity was mapped by first producing all possible pairwise Sørensen values between regions and then performing a two-dimensional non-metric multidimensional scaling ordination of the results. Regions were then assigned colours according to their location in non-metric multidimensional scaling ordination space based on the Hue-Chroma-Luminance (HCL) colour scheme shown in actually Fig. 3.

### Bioregionalization

To define the most natural delineation within the passerine distribution data we assessed the clustering performance of nine different clustering algorithms (listed in the supplementary material of Holt *et al*.^26^) for every possible number of clusters (2–31). For each possible number of clusters we followed this procedure:

1. Produce a result for each clustering algorithm.

2. The performance for each clustering result was quantified by calculating the percentage of the sum total of PhyloSor values that were between, rather than within, clusters. This evaluation metric is referred to as ‘explained variance'. Because different clustering results have different numbers of locations within clusters and different total numbers of clusters, they cannot be reliably compared by using these empirical explained variance values.

3. Standardised effect sizes (SES's) were calculated for each empirical explained variance value, to allow for comparison of all of the clustering results. Expected explained variance was calculated for all clustering results by calculating the explained variance for the same clustering on a randomized null distributional data set. To produce these null communities, species occurrences were randomly shuffled across the distributional data using the Miklós and Podani^27^ algorithm, which maintains the number of species per location and the number of occurrences per species. This process was repeated 1,000 times and the SES explained variance was calculated as:


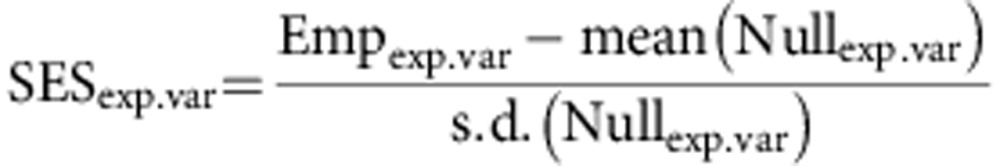


Whereby Emp_exp.var_=empirical explained variance and Null_exp.var_=null explained variance.

4. The clustering result that produced the highest standardized effect size was used as our bird distribution data for the main analysis. Clusters of locations are referred to as ‘realms'. Since this clustering result failed to resolve island and continental areas, the clustering process was repeated within realms to subdivide them into ‘regions'.

### Detecting local speciation, import and export

Biogeographical source/sink dynamics were examined by using ancestral area reconstruction analysis, which assigned putative regions for ancestral nodes in the phylogeny. Ancestral areas were assigned to each node based on maximum likelihood ancestral discrete character estimation using the ‘ace' function in the R package ‘ape'^28^ (Supplementary Fig. 2). In situations where an extant species occurred in more than one region, a new discrete character state was created by combining the regions concerned. An ancestral node was assigned to a region if the probability of such an assignment exceeded 75%, with all ancestral nodes not showing this level of probability remaining unassigned. In addition we ran all analysis using alternative cutoff values of 50 and 95%.

‘*In situ* speciation events' were identified as cases in which ancestral nodes were assigned to the same location as a descendent node. ‘Export' events were defined as cases where the region of a descendent node (either a tip or an internal node) is different from the region of the focal ancestral node. In the reverse situation, ‘import' events were defined as cases where a focal descendent node (either a tip or an internal node) was assigned to one region and its ancestor assigned to a different region. In all cases, we refer to a ‘node' as being either a tip (that is, extant species) or an internal node (an ancestral species). Note that not all internal nodes were successfully assigned to a region, and that both ancestor and descendent need to be assigned to a region for an export, import or *in situ* speciation event to be detected.

### Comparison of empirical data with null models

Whether these results are simply an artefact of contemporary diversity patterns cannot be determined by simply studying these empirical results and therefore a comparison with null results that account for these distributional patterns is necessary.

We designed null models to generate random expectations and ask: given present distributions of passerine species across the globe, how many evolutionary transitions are expected between regions? Our null model fixed global patterns of species diversity (that is, species richness (Fig. 3a) and species turnover (Fig. 3b)) and randomly shuffles the species across the phylogeny to simulate a situation in which the evolution of passerine birds has not been geographically restricted within any part of their overall current distribution. This null-model design treats all regions equally and returns expected numbers of transitions between regions given contemporary distributional patterns. Results were generated for 1,000 of these null runs (one randomly shuffled (null) tree is provided in Supplementary Fig. 3), based on the same analysis described for the empirical patterns, to provide mean null expectations for numbers of evolutionary transitions (Fig. 4, grey arrows). We then compare the empirical result with these null expectations by calculating standardized effect sizes of the number of transitions between regions (Fig. 5). Standardized effect sizes were generated for each region for five biogeographical parameters: (1) total dispersal (that is, export plus import), an indication of how connected (or how isolated) a region is; (2) net dispersal (that is, export minus import), which indicates whether a region generally acts as a source or a sink; (3) export; (4) import; and (5) *in situ* speciation (that is, generation of evolutionary lineages within the region). In all cases we refer to ‘dispersal' in terms of evolutionary lineages, rather than in terms of ecological dispersal of individuals. To investigate the impact of species that have been added to the phylogeny based only on taxonomy (that is, species with no genetic data), we repeated our analysis on 100 randomly selected data-only trees (4,032 species) from the pseudo-posterior distribution^13^. On each of these 100 trees we generated 100 null expectations (summarized in Supplementary Fig. 1).

## Additional information

**How to cite this article:** Jønsson, K. A. & Holt, B. G. Islands contribute disproportionately high amounts of evolutionary diversity in passerine birds. *Nat. Commun.* 6:8538 doi: 10.1038/ncomms9538 (2015).

## Supplementary Material

Supplementary Information Supplementary Figures 1-3

## Figures and Tables

**Table 1:** Dispersal between and speciation within regions.

**FromTo**	**IM**	**Or**	**Au**	**PP**	**Oc**	**NZ**	**PN**	**NT**	**NA**	**Ca**	**PL**	**Ha**	**Af**	**Ma**
IM	532	50	11	19	19	0	0	0	0	0	3	0	10	1
Or	79	808	0	0	0	0	2	0	5	1	28	1	10	0
Au	5	1	371	28	7	1	0	0	0	0	1	0	0	0
PP	17	4	24	384	10	3	0	0	0	0	0	0	3	1
Oc	20	3	14	16	251	1	0	0	0	0	0	1	1	0
NZ	0	0	0	0	0	10	0	0	0	0	0	0	0	0
PN	0	0	0	0	0	0	155	9	13	7	0	1	0	0
NT	1	1	0	0	0	0	273	3,535	18	33	1	0	1	0
NA	1	0	0	0	1	0	29	8	258	11	3	0	0	0
Ca	0	0	0	0	0	0	6	4	7	80	0	0	0	0
PL	9	51	1	2	6	0	7	1	14	0	629	0	33	2
Ha	0	0	0	0	0	0	0	0	0	0	0	36	0	0
Af	38	66	6	8	4	1	6	6	9	1	68	0	2,217	21
Ma	1	3	0	0	1	0	0	0	0	0	0	0	2	118

Af, African; Au, Australian; Ca, Caribbean; Ha, Hawaiian; IM, Indo-Malayan; Ma, Madagascan; NA, Nearctic; NT, Neotropical; NZ, Novo-Zelandic; Oc, Oceanian; Or, Oriental; PL, Palaearctic; PN, Panamanian; PP, Papuan.

Evolutionary transitions and ‘*in situ* speciation' events between and within biogeographical regions based on ancestral area reconstruction. Phylogenetic node assignments based on 75% probability cutoff values.
