# Supplementary material for: Islands contribute disproportionately high amounts of evolutionary diversity in passerine birds
Source: Nat Commun. 2015 Oct 5;6:8538. doi: 10.1038/ncomms9538 (PMC4600755; doi:10.1038/ncomms9538)
Supplement: Supplementary Information — Supplementary Figures 1-3 [file ncomms9538-s1.pdf]

Supplementary Figure 1

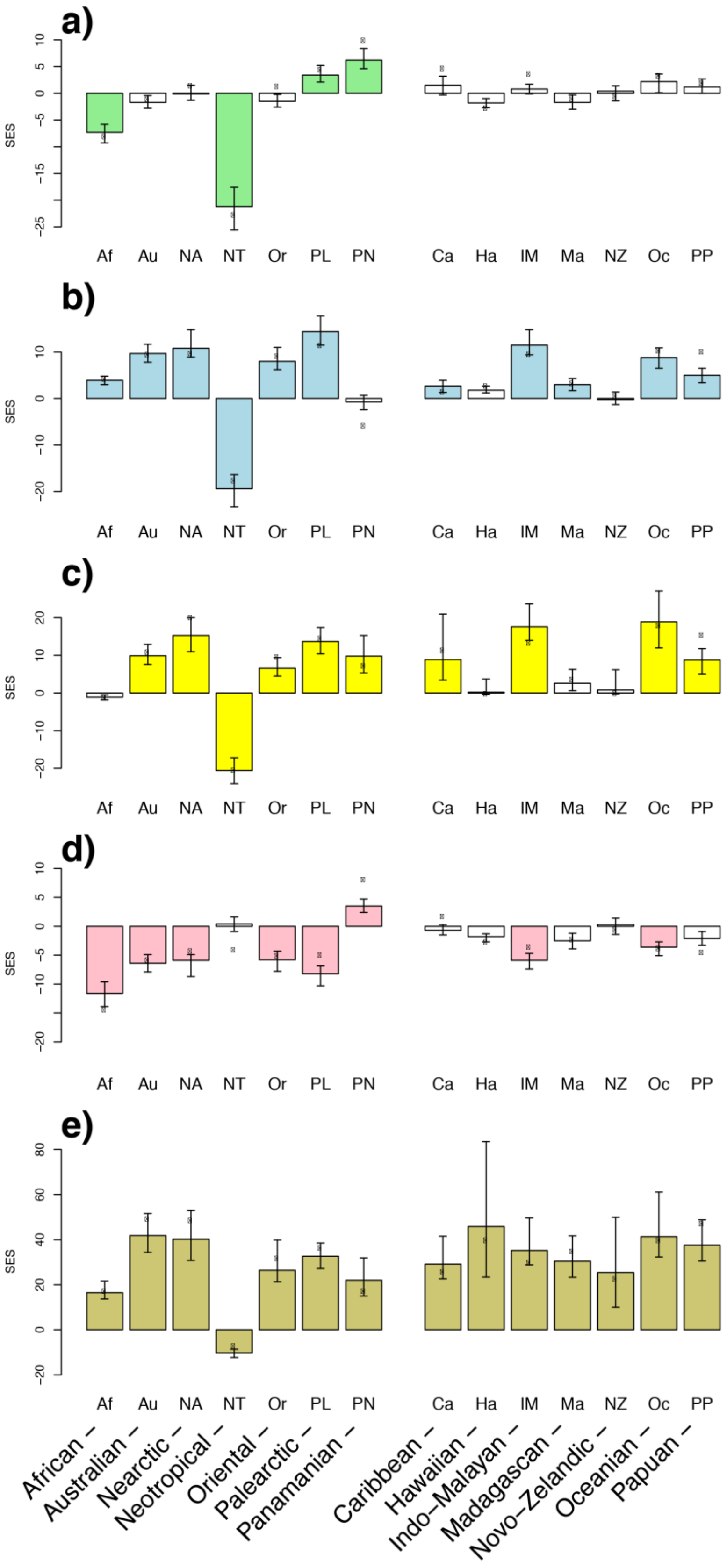

Supplementary Figure 1 Regional dispersal and speciation relative to the expected based on 100 data-only trees

Bar plots indicate various dispersal metrics compared to the null expectations derived from randomisations constrained to account for existing diversity patterns (i.e. Standardised Effect Sizes). From the top: a) total dispersal (import and export combined), which is an indication of how dynamic the region is, b) net dispersal (export of species minus import of species), which is an indication of whether the region is overall a source or a sink, c) export, d) import and e) diversification within regions. This figure is based on 100 randomly selected data-only trees (4,032 species) from the pseudo-posterior distribution[12]. On each of these 100 trees we generated 100 null expectations). Bar heights reflect mean standardised effect sizes and error bars reflect 95% quantiles around the mean. Points represent results from the full analysis (i.e. using the phylogeny that included species with no data), which is shown in the main manuscript as in Fig. 5.

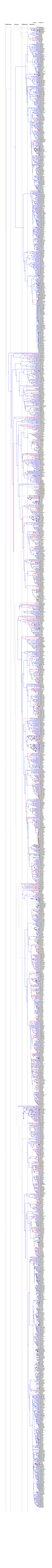

Paleocene
Eocene
Oligocene
Miocene
Pliocene
Quaternary

Supplementary Figure 2 Ancestral areas of data-only passerine bird tree

Tree depicting the data-only consensus tree[12] with the ancestral areas depicted for each node according to the 75% threshold. Blue lineages represent *in situ* speciation events, red lineages represent dispersal events and grey lineages are omitted from the analysis because a given area cannot be assigned with more than 75% probability.

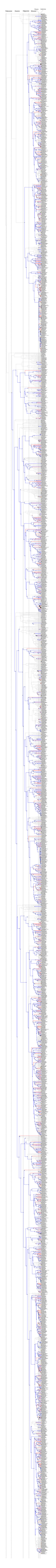
Pliocene
Quaternary
Paleocene
Eocene
Oligocene
Miocene

Supplementary Figure 3. Ancestral areas of randomly shuffled (null) data-only passerine bird tree

One randomly shuffled (null) tree with the same tip names as in Fig. S4 with the ancestral areas depicted for each node according to the 75% threshold. Blue lineages represent *in situ* speciation events, red lineages represent dispersal events and grey lineages are omitted from the analysis because a given area cannot be assigned with more than 75% probability.
